# Squat Kinematics Analysis Using Vicon and Affordable Motion-Capture Solutions

**DOI:** 10.3390/s25113294

**Published:** 2025-05-23

**Authors:** Urszula Czajkowska, Michał Popek, Celina Pezowicz, Bogna Leśnik, Magdalena Żuk

**Affiliations:** Faculty of Mechanical Engineering, Wroclaw University of Science and Technology, Wybrzeże Wyspianskiego 27, 50-370 Wrocław, Poland; mpopekwroc@gmail.com (M.P.); celina.pezowicz@pwr.edu.pl (C.P.); 261088@student.pwr.edu.pl (B.L.); magdalena.zuk@pwr.edu.pl (M.Ż.)

**Keywords:** virtual reality, HTC Vive Trackers, kinematic measurement

## Abstract

The analysis of human movement is crucial in biomechanical research and clinical practice. Quantitative movement analysis evaluates sports performance by tracking joint angles, segmental velocities, and body positions. There are high-accuracy motion-tracking systems like Vicon Motion Systems (Oxford, UK) or OptiTrack (Corvallis, OR, USA), but they are expensive, require expertise, and lack portability. This study assessed a low-cost virtual reality-based motion-tracking system with a customized eMotion data acquisition and analysis application to describe joint movements during squatting. The system, which utilizes commonly available virtual reality accessories, successfully collected kinematic data and continuous tracker trajectories. The results showed high repeatability comparable to advanced optoelectronic motion-capture systems. The eMotion system protocols exhibited low variability for most rotations, with inter-trial values ranging from 0.65° to 2.20° except for hip and knee flexion, which reached 3.09° and 4.01°. The motion-tracking technology that is part of VR headsets has great potential in supporting training and rehabilitation by enabling quantitative assessment of any activity in both the real and virtual worlds. The use of low-cost solutions can increase the potential for human motion measurements in clinical practice and biomechanical research.

## 1. Introduction

Virtual reality (VR) is a technology that is gaining popularity and finding more and more applications, a comprehensive analysis of which can be found in the work of Hamad and Jia [1]. A promising area of VR applications is sports and rehabilitation [2]. Studies have shown the beneficial effects of using this technology in therapy to achieve various goals, including for pain relief, muscle strengthening, increasing the joint range of motion, and improving the functional ability and motivation of patients [2,3,4]. The most popular VR headsets include base stations, goggles, and handheld controllers. These devices enable the body and objects to be visualized in a virtual environment and for natural and intuitive interactions with environmental elements to take place. Moreover, virtual reality accessories enable the collection of quantitative data describing the user’s motor tasks by tracking joint angles, segment velocities, and body positions [5,6,7].

There are many tracking systems available on the market. Marker-based motion-capture systems are considered the gold standard for the three-dimensional kinematic measurements of joints. This system uses reflective markers placed on anatomical landmarks, as recommended by the International Society of Biomechanics (ISB), to capture their spatial trajectories [8]. However, these marker-based systems have some limitations, such as the high cost of equipment, the time-consuming attachment of markers, and the need for technical expertise. As a result, their practical application is mainly limited to specialized institutions [9,10].

Another type of tracking system is the use of Inertial Measurement Units (IMUs), which are widely used for human motion analysis due to their portability, cost-effectiveness, and ability to operate without external infrastructure. These systems typically consist of accelerometers, gyroscopes, and, sometimes, magnetometers, enabling real-time motion tracking in a variety of environments. One of the main advantages of IMUs is their ability to capture motion outside of laboratory conditions, enabling their application in sports science or ergonomics.

However, IMUs also have some limitations. One major challenge is signal drift, particularly in gyroscopes, which can lead to accumulated errors over time and affect the accuracy of motion reconstruction. Unlike optical systems, IMUs do not provide absolute position tracking, making them less suitable for capturing global motion trajectories [11,12,13,14].

Another group of tracking solutions consists of inexpensive systems designed to be accessible to a wider audience. These include HTC Vive Trackers, Microsoft Kinect, and video systems such as Kinovea and OpenCap [6,10,15,16,17,18]. Microsoft Kinect technology has found applications in various fields, including healthcare, education, robotics, sign language recognition and 3D reconstruction [19]. Despite the fact that Microsoft discontinued Kinect in October 2023, there have been continuous publications describing practical applications of the device [20,21,22]. Virtual reality accessories are currently gaining interest as components of motion-tracking systems [10,23,24,25]. Their availability, relatively low cost, and ease of implementation make them suitable not only for scientific research but also for clinical, rehabilitation, sports, and educational applications. The development of these technologies enables the creation of more affordable and scalable motion-analysis systems, which is essential for making biomechanical methods more widely accessible beyond specialized laboratories. However, these systems currently face limitations in reliability and accuracy, which constrain their application in high-precision contexts. Ongoing refinement of tracking and calibration algorithms is therefore crucial to improving their performance and unlocking the full potential of precision motion monitoring in areas such as medicine and interactive virtual reality technologies.

The HTC Vive Trackers, developed by HTC (New Taipei City, Taiwan), are small tracking accessories designed to work with the HTC VR headset. They are becoming popular as a ready-made, low-cost, room-scale tracking system and consist of two infrared-emitting Lighthouse 2.0 base stations and trackers equipped with mounted photodiodes. The base stations emit structured infrared light, which allows the system to determine the position of the trackers in 3D space. The trackers also use motion data from their own inertial measurement unit (IMU) to maintain continuous measurements when the optical tracking system’s line of sight is obstructed [26]. Their precision has been shown to vary depending on the placement of the Lighthouse 2.0 and the speed and type of recorded motion [6,16,17,18,19,20]. It is not clear what tracking quality can be obtained when using these accessories to evaluate how physical exercises are performed (within or outside the VR environment) due to their variability, complexity, and varying performance speeds.

Motion assessment is associated with measurement protocols that define anatomical coordinate systems, calculate joint angles, and provide instructions for marker placement [27]. The conventional gait model (CGM) is the most commonly used biomechanical model in clinical gait analysis and has been popularized by Vicon’s clinical motion-capture software packages (e.g., Vicon Nexus 2.12; Vicon Motion Systems, Oxford, United Kingdom) [28].

As part of a project to develop a computer-aided training and rehabilitation system using VR and motion-capture technology called eMotion, a custom application was proposed to acquire data from HTC VIVE devices (goggles, controllers, and Vive Tracker sensors; HTC Corporation, New Taipei City, Taiwan) and to analyze the collected results. When recording positional data from HTC Vive Trackers using the eMotion system, the coordinates of the origin of each tracker’s local reference frame are tracked relative to the global coordinate system. In addition to position, the system also provides orientation, which describes the rotation of the local tracker system relative to the global one. This origin is located in the mounting thread at the bottom of the device. Joint angles were computed as a sequence of three Cardan angles (flexion/extension, adduction/abduction, internal/external rotation) describing the relative orientation between adjacent segments. The hip-joint center was estimated using the predictive method by Bell et al. [29], implemented in custom software. The measurement protocols implemented in the system included ISB 6DOF and Simplified 6DOF. The ISB 6DOF protocol requires anatomical calibration and establishes coordinate system definitions according to the recommendations of the International Society of Biomechanics (ISB). As part of this protocol, specific anatomical landmarks (listed in the Section 2) are identified to define the segmental anatomical coordinate systems used in joint kinematic calculations. It is worth noting that in the ISB6DOF protocol, the foot coordinate system is defined by virtual shank markers placed in the neutral foot position, while joint angles are calculated based on the tracker’s orientation as described in Żuk et al. [25]. Additionally, auxiliary points can be designated to support various aspects of motion analysis, including enhanced movement animation, detection of gait events such as heel strike, calculation of temporal gait parameters, and improved scaling of musculoskeletal models for use in dynamic simulations [30].

In contrast, in the simplified 6DOF protocol, the coordinate system of each body segment is assumed to be identical to that of the corresponding tracker, thus eliminating the need for additional calibration. In the simplified 6DOF protocol, each tracker is assigned to a single body segment, and it is essential to align it as closely as possible with the segment’s anatomical axis. An exception applies to the tracker placed on the foot, whose precise location is not critical due to the use of a position-correction method. In this approach, during a static standing posture with feet positioned parallel, the ankle-joint angles are assumed to be zero. A detailed description of these protocols was presented in an earlier paper [25], which also validated the proposed tools for an example of gait analysis. The present study aimed to evaluate the possibility of using a low-cost eMotion motion tracking system to describe joint kinematics during squatting, a basic human movement incorporated into many everyday activities [31], and to verify and validate the methods and tools proposed in the article by Żuk et al. [25]. The objectives were achieved through a kinematic analysis of the squat in a group of young healthy subjects, along with an analysis of the repeatability of joint-angle measurements obtained using three protocols: Simplified 6DOF, ISB 6DOF (recorded and calculated with the eMotion system), and the Plug-in Gait model (with a Vicon system).

## 2. Materials and Methods

Ten healthy, injury-free adults (4 women and 6 men) were analyzed (mean age: 24 ± 4 years; mean height: 172.5 ± 10.5 cm; mean mass: 66.7 ± 12.07 kg). The local Ethics Committee approved the study, and all participants provided their written informed consent before participating.

**Figure 1:**
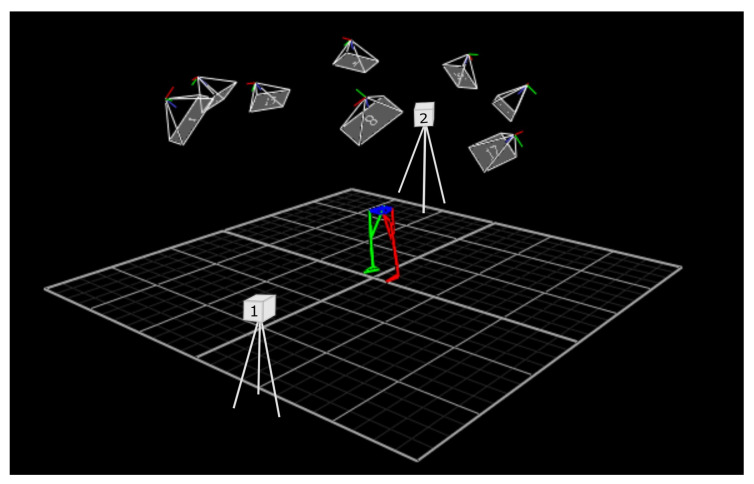
Study area schematic generated with Nexus software, with overlaid VR “Lighthouse” positions.

An eight-camera motion-capture system (Vicon Motion Systems Ltd., Centennial, CO, USA; sampling frequency: 100 Hz) with Nexus software (version 2.15; Vicon Motion Systems Ltd., Oxford, UK) and seven HTC Vive Tracker 3.0 devices were used to record the kinematics of the joints of the lower extremities, along with two Lighthouse 2.0 base stations of the HTC Vive Pro Full Kit (HTC, New Taipei City, Taiwan) placed 5.7 m apart. All sensors were connected to a ROG Strix G15 laptop (Asus, Taipei, Taiwan) using an 8-port USB 3.0 charger hub (iTec, Ostrava, Czech Republic). During the test, a subject stood at the center of the measurement space, as shown in Figure 1.

The subjects were prepared for the measurements by placing Vive Trackers on their lower extremities using Vortex attachment straps. The sensors were attached to the following locations: pelvis (on the right side), thighs (lateral), shins (distally, lateral), and feet (dorsal side). Passive markers with a diameter of 10 mm, dedicated to the Vicon system, were then placed on each subject’s body according to the Plug-in Gait protocol on the following anatomical landmarks: LASI (left anterior superior iliac spine), RASI (right anterior superior iliac spine), LPSI (left posterior superior iliac spine), RPSI (right posterior superior iliac spine), LTHI (left thigh), LKNE (left knee), LTIB (left tibia), LANK (left ankle), LHEE (left heel), LTOE (left toe), RTHI (right thigh), RKNE (right knee), RTIB (right tibia), RANK (right ankle), RHEE (right heel), RTOE (right toe).

**Figure 2:**
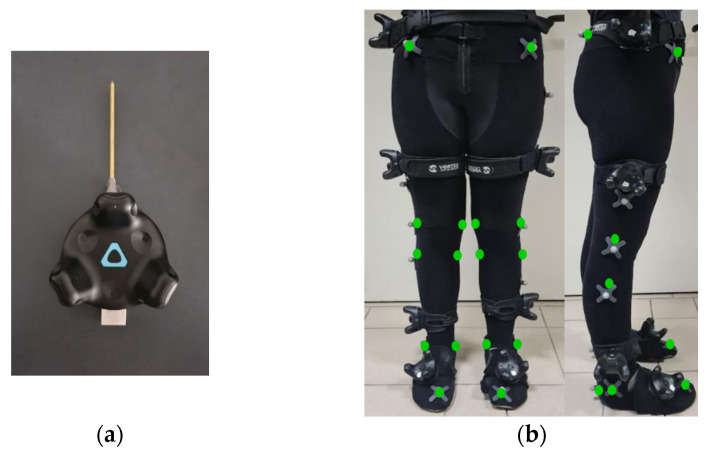
(**a**) A tool with a navigable tip mounted on a tracker for the anatomical calibration procedure; (**b**) Position of trackers (eMotion system), reflective markers (Vicon system), and virtual anatomical points (green color) used for calibration according to the ISB 6DOF protocol during the experiments, shown for a representative participant.

Before the actual measurements, calibration was performed, during which 19 anatomical points were defined using a navigation pointer consisting of an HTC Vive Tracker 2.0 and a mounted 16 cm long steel tip (Figure 2a). The defined points were the anterior superior iliac spines, the midpoint between the posterior iliac spines, the medial and lateral epicondyles of the femurs, the medial and lateral condyles of the tibias, the lateral and medial ankles, the calcaneal tuberosities, and the first metatarsal bones. The 19 virtual anatomical points calibration takes a few minutes. The use of this protocol makes it possible to obtain data on the trajectories of the virtual points placed on characteristic anatomical landmarks, allowing for dynamic simulations, which is a significant advantage. The locations of the sensors and markers are shown in Figure 2b. A detailed description of the pointer and anatomical calibration process, as well as a custom eMotion software module for data acquisition, was provided in an earlier article [25].

**Figure 3:**
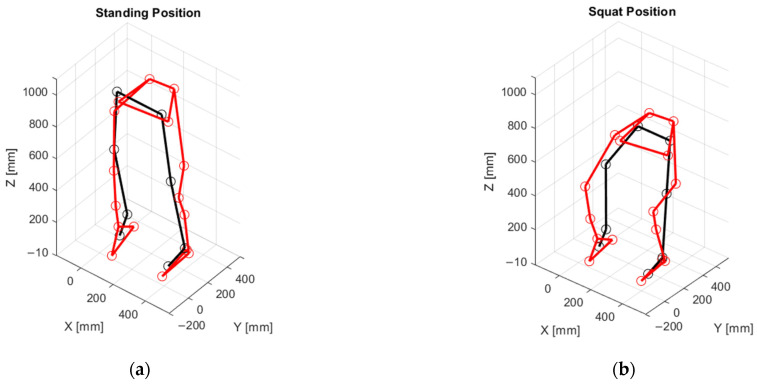
3D skeletal model reconstruction diagrams at critical motion nodes for a representative participant: (**a**) standing position; (**b**) lowest squat position. Black: HTC Vive Trackers; Red: Vicon markers. Calibration and marker placements were individually adjusted for each subject.

After recording a static position using the two systems, each subject performed seven squats with their feet hip-width apart and parallel, as presented in Figure 3. Subjects were not given precise instructions on exercise technique and could choose their own pace. The five middle repetitions were selected for analysis. The data for all three models—two based on the eMotion system (using the Simplified 6DOF and ISB 6DOF protocols) and one using the Plug-in Gait model with the Vicon system—were recorded simultaneously. Model differentiation and joint-angle calculations for the eMotion system were performed during the post-processing stage using a custom-built analysis application, which processes HTC Vive Tracker data according to the selected method. Diagrams illustrating the 3D skeletal model reconstruction during free standing and at the critical point of motion, corresponding to the lowest squat position, are presented in Figure 3. The eMotion system utilizes a single sensor placed on the right side of the pelvis; for visualization purposes within the MATLAB (R2022b version) environment, a symmetrical virtual sensor was created on the left side to enable the representation of both lower limbs in a model visualization.

### The Data Analysis

During the data analysis, two repetitions were excluded due to incomplete data recording, resulting in a final analysis of 48 squat repetitions performed by 10 subjects. All the data processing and analysis were performed in MATLAB (R2022b version). The recorded data were first segmented to isolate individual squat repetitions for each participant. Segmentation was based on identifying the midpoint of the standing position, defined by the minimal values of knee and hip flexion. These points were used as cutting points to extract separate movement cycles, which were then time-normalized to enable consistent analysis across repetitions and subjects.

**Figure 4:**
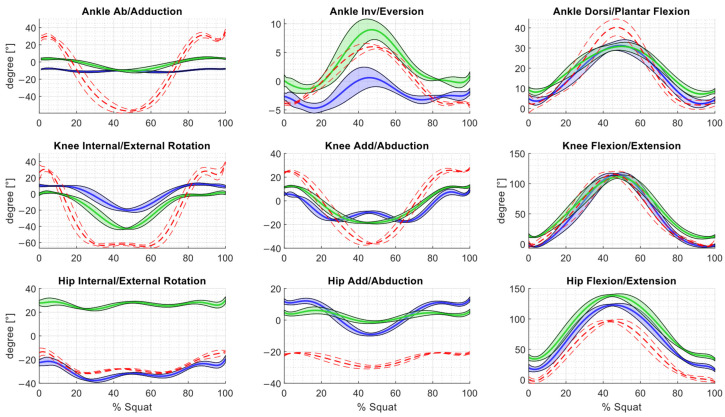
Kinematic variables calculated by the three methods, averaged across five squat cycles of a representative subject. Blue: eMotion + Simplified6DOF (±SD); green: eMotion + ISB6DOF (±SD); red: Vicon + Plug-in-Gait Model (±SD).

The analysis was performed for movements in three planes (flexion/extension, abduction/adduction, internal/external rotation) for the joints of the right lower limb (dominant in each of the subjects). Five repetitions (in two cases, four) were selected for each person, normalized to 100% (each measurement was resampled to 100 frames, representing 100% task completion), and then the mean value and standard deviation were calculated. The angular curves for each subject were calculated as the mean of the five repetitions, and the repeatability of the measured kinematics was assessed using the method described by Schwartz et al. [32]. The average inter-trial variability (AIT) was computed and compared with values reported in recent studies [25,31,32] according to the following steps:

1. Average joint-angle calculation (per participant):

For each participant, calculate the average value of the analyzed joint-angle variable across five repetitions of the task for each frame in the measurement sequence. Figure 4 illustrates the changes in values of the joint angles in the lower limb during a squat for a sample individual.

2. Standard deviation calculation (per participant):

For each participant, calculate the standard deviation of the joint-angle variable across the five repetitions for each frame.

3. Repeat for all participants:

Apply steps 1 and 2 to all participants individually.

4. Average standard deviation across participants (per frame):

For each frame, compute the mean of the standard deviation values obtained from all participants. This results in a time series showing the group-level variability throughout the movement.

5. Overall inter-test variability (single value for the group):

Calculate the average of the frame-by-frame values obtained in step 4. This results in a single numerical value representing the overall inter-test variability for the entire group.

To assess the differences in sampling variability between the experimental protocols, we conducted an F-test, treating the measured variability as the dependent variable. Before proceeding with the analysis, the assumptions of normality and homogeneity of variance were verified using the Shapiro–Wilk and Levene’s tests, respectively. Additionally, the range of motion for all joints was calculated, and differences between each pair of protocols were analyzed. It should be noted that signal gaps in the Vicon data occurred at the lowest point of the squat movement due to the occlusion of markers placed on the anterior superior iliac spines (ASISs), which affected up to 10% of the recorded signal.

## 3. Results

The Vive Tracker sensors allowed for full data collection without gaps. In the case of the Vicon optical system, the markers were not visible during the middle squat phase. To analyze the joint kinematics, interpolation had to be performed (Matlab, spline function). Figure 4 presents the angular curves of a representative subject, calculated as the mean of five sets (including the standard deviation).

**Figure 5:**
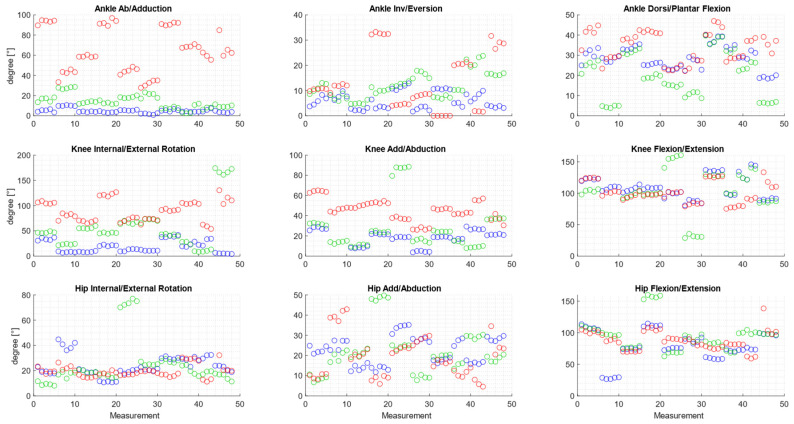
Joint range of motion (ROM) values calculated for each individual trial. Blue: eMotion + Simplified6DOF (±SD); green: eMotion + ISB6DOF (±SD); red: Vicon + Plug-in-Gait Model (±SD).

Comparing the Plug-in Gait model (with Vicon system) with the eMotion’s system protocols, it can be seen that the plotted characteristics differ, especially for ankle abduction/adduction and knee internal/external rotation. As shown in Table 1, the range of motion (ROM) values at each joint vary depending on the measurement method and protocol used. The values of the calculated standard deviation are also different. The results of the ROM measurements for the individual trials are presented in Figure 5.

Four to five repetitions were analyzed for each participant, with most trials yielding similar ROM values, as evidenced by the clustering of points in Figure 5.

The differences between ROM values calculated using the different protocols are posted in Table 2. The largest differences between the results were observed for knee rotation (up to 70.7° ± 21.5° when comparing ISB 6DOF with trackers to the Plug-in Gait model) and ankle abduction/adduction, where the difference was as high as 60.3° ± 22.5°.

**Figure 6:**
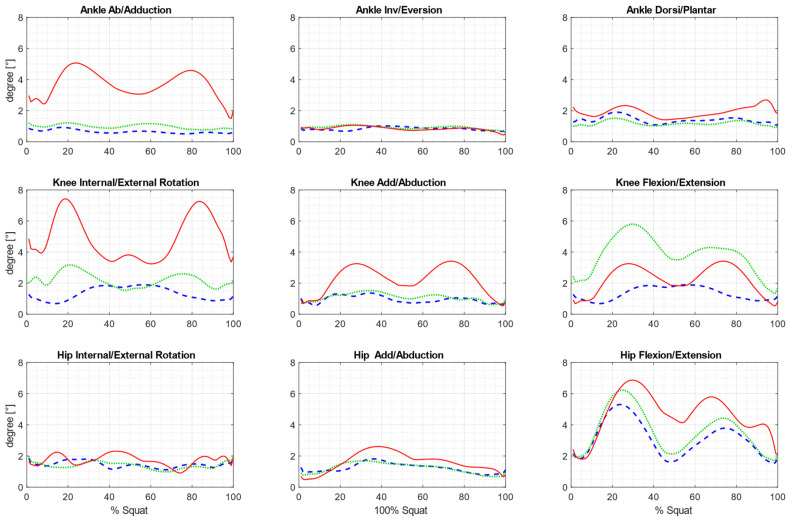
Patterns of inter-trial variability across all samples of the squat cycle, averaged for all subjects. Blue: eMotion + Simplified6DOF ± SD; green: eMotion + ISB6DOF ± SD; red: Vicon + Plug-in Gait Model ± SD.

The repeatability of the measured kinematic parameters is illustrated in Figure 6 as a pattern of inter-trial variability across all samples of the squat cycle, with its value varying according to the phase of the motion.

In the case of the eMotion system protocols, the average variability is low for most rotations (Table 3) and similar to the corresponding data from other studies [25,32,33]. However, it should be noted that the cited papers refer to the analysis of gait kinematics. The inter-trial values for the Simplified 6DOF protocol ranged from 0.65° to 1.45° for most of the joints except for hip and knee flexion (where they reached 3.09° and 4.01°, respectively). The values obtained using the ISB 6DOF protocol ranged from 0.99° to 2.20°, except for movements such as in the previous protocol, where they reached 3.83° for knee flexion and 3.58° at the hip. The results obtained using the Vicon system were similar to those reported by other authors for hip flexion, hip adduction/abduction, knee adduction/abduction, ankle inversion/eversion, and ankle abduction/adduction [25,32]. However, the AIT for the Plug-in Gait model exceeded 3.22° for other rotations, reaching 4.91° for knee flexion. The F-test showed statistically significant differences in the variability between samples for 16 of the 27 comparisons (Table 4).

## 4. Discussion

An analysis of the repeatability and functionality of two motion analysis systems was performed to assess squat kinematics. Data were collected using two systems: a multicamera optoelectronic system from Vicon Motion Systems and a system based on HTC Vive Tracker sensors combined with dedicated custom eMotion software for data acquisition and analysis (2.8.0.0 version).

The Vicon system requires the precise placement of passive markers on the subject according to the Plug-in Gait protocol for measurements. The way the markers are placed affects the results describing the kinematics of the joints. It is also important to note that in practical applications, markers are often affixed to the skin of the subject or attached to a specialized suit with Velcro fasteners [34]. Due to gaps in the data acquired from the Vicon system, interpolation was necessary, which may have introduced a potential source of bias into the results. A common application of multicamera systems in clinical practice is gait analysis [28,35,36], where the risk of data gaps is lower, but in the analysis of motor tasks such as squats, bends, or lunges, the risk of marker obstruction increases. During the current measurements, visibility problems were observed with markers placed on the front upper hip spines, which were temporarily obscured during squatting due to their position between the thighs and torso. For these reasons, the use of these systems to assess the quality of exercise performance in rehabilitation or sports practice is limited. This challenge could be partially mitigated by increasing the number of cameras or adjusting their location, for example, lowering them, but this would also lead to higher system costs.

The tracking system based on VR accessories did not cause any problems in terms of the calibration of the measurement space. The applied methods, using the eMotion system, allowed for continuous data collection without gaps. The components are portable and easy to use. The proposed solution is affordable. The price of a set for analyzing the kinematics of both lower limbs, which includes one lighthouse and seven sensors, is approximately $1000.

While the cost of the proposed VR-based tracking system may be comparable to IMU-based approaches, its primary advantage lies in its ability to provide high-precision, real-time 3D tracking without the need for complex sensor calibration or the risk of drift, which are common issues in IMU systems. Furthermore, the VR accessory-based system combines the advantages of both optoelectronic and IMU systems, allowing their respective capabilities to complement one another. Additionally, this system can serve as a valuable enhancement to VR applications for rehabilitation or training, offering real-time feedback and an interactive environment to help users improve their fitness or recovery.

The simplified 6DOF protocol only required careful placement of the trackers on the subject’s body. This process was not difficult, but it could have potentially impacted the alignment of the coordinate systems, leading to shifts in the results. In the case of the ISB 6DOF protocol, there was a need for calibration, which required knowledge of the location of specific anatomical landmarks. A challenge encountered during the study was the potential movement of the Vive Trackers from their original positions, which could have influenced the obtained results. In the graphs showing three curves representing changes in the joint-angle values calculated using different protocols, it is evident that although the overall shape of the curves is similar, they exhibit noticeable vertical offsets. This effect is particularly pronounced in the hip joint, especially for rotational movements, where one curve begins at around 30°, while the others start between 0° and –20°. These discrepancies are likely attributable to differences in the definition of the local coordinate systems assigned to each body segment. To enable consistent comparison between datasets, it may be advisable to apply zero-offset corrections based on joint-angle values recorded in a neutral standing position (i.e., upright posture with fully extended and unrotated lower limbs).

One factor that may also affect the accuracy of the proposed method is the variability of participants’ physiological characteristics, which was not included in the analysis. Differences in parameters such as height, BMI, or limb length can significantly affect movement dynamics and tracking accuracy [37,38,39]. Possible sources of error include individual differences in body proportions, which can lead to different kinematic patterns. In future research, it would be valuable to consider the influence of participants’ physiological characteristics—such as height, BMI, and limb length—on movement dynamics and tracking accuracy, as these factors were not included in the present analysis.

Comparison of joint kinematics during selected motor tasks is commonly used in research. However, scientists adopt different methods to compare the results. Das et al. compared lower-limb joint kinematics during lunges that were obtained using a markerless and marker-based motion tracking system, demonstrating that the markerless motion-capture system overestimated the kinematic measurements for most angles [8]. Kristiansen et al. examined inter- and intra-individual variability in squat kinematics, demonstrating the influence of anthropometric factors and movement strategies on the consistency of the results [40]. Erman et al. analyzed the effects of fatigue on the kinematics of the bodyweight squat, finding significant changes in joint movements and velocity patterns [41].

Repetition measurement is a valuable tool for evaluating technique in exercise, including measuring parameters such as joint angles or speed. This approach provides insight into the stability of movement tasks, the impact of fatigue, and emerging movement patterns. Even highly skilled individuals exhibit movement variability when performing the same goal-orientated task over multiple repetitions [42,43,44,45,46]. The repeatability of measured joint kinematics, assessed through inter-trial variability analysis, is a widely used method in biomechanical research [16,40,47,48,49,50]. In our study, we analyzed the inter-trial variability during the evaluation of the squat, interpreting it as a measure of movement variability. The use of three different methods for calculating kinematics allowed us to compare them with each other, with a particular focus on the comparison of results between the low-cost and the gold-standard tracking system.

These results were consistent with the repeatability values reported by other authors, although their studies focused on walking rather than squatting [25,32,33]. By comparing the AIT values with those from previous studies, we can evaluate the effectiveness of the new tracking system in obtaining reproducible measurements using biomechanical protocols adapted to it, compared with existing solutions. The AIT value for the Simplified 6DOF protocol was 3.09° for hip flexion and 4.01° for knee flexion, whereas for the ISB 6DOF protocol, these values were 3.53° and 3.83°, respectively, which exceeded those reported by other authors for these rotations. However, it should be noted that human gait is characterized by high repeatability, with smaller angular changes in the joints during the gait cycle. In contrast, during squats, variability in the achieved range of motion seems unavoidable, particularly in joints where the movement occurs over a wide range (such as hip and knee flexion in squats). The AIT values obtained with the Plug-in Gait model for eight rotations exceeded the values calculated with the Vive Trackers. Only for hip flexion was the value lower at 1.73°.

Discrepancies between the results obtained by different methods may be due to the different orientations of the coordinate systems of the body segments as well as the movement of the sensors and markers during the test [51,52,53]. One of the key areas for potential improvements in the HTC Vive Trackers’ motion-tracking system is in enhancing the stability and precision of sensor mounting. Sensor displacement during movement can lead to errors in calculating joint angles; therefore, implementing ergonomic mounts with better stability, such as reinforced straps or self-adjusting bands, could significantly improve measurement accuracy. Another enhancement could involve developing a position-compensation algorithm, which would determine the actual relative positions of the trackers based on an initial calibration. This algorithm could perform a zeroing procedure, where the user assumes a predefined reference position (e.g., standing in a neutral anatomical stance), which would allow for the correction of potential errors caused by uneven sensor placements. The calibration process could also account for individual anatomical differences, leading to more accurate joint-angle representation and increased reliability in motion analysis.

Combining optimized sensor mounting with intelligent calibration could significantly improve the measurement repeatability and accuracy, making the system more resilient to errors caused by sensor displacement and individual user characteristics.

Vox et al. reported that when comparing joint-angle measurements obtained with Vive Tracker sensors and Final IK software to those calculated using the Qualisys system, joint-angle deviations ranging from ±6° to ±42° were observed [6]. In our study, for each participant, four to five repetitions were analyzed. The ROM plots (Figure 5) show that while the results obtained using the different methods may vary in magnitude, the values within individual participants tend to cluster, suggesting a degree of consistency across repetitions. This pattern indicates that observed differences are more likely related to inter-method calibration discrepancies than to variability in the performed movements. Notably, the characteristics of changes in joint angles differ noticeably between the eMotion system and the Vicon system. This is particularly true for inversion/eversion and abduction/adduction in the foot as well as for knee rotation. This is consistent with reports by other authors, who observed that the kinematics of movements in the sagittal plane was comparable between different protocols, while significant differences appeared in the transverse and frontal planes [54,55]. Of particular note is the case of foot inversion/adduction, where the Vicon system recorded a ROM value of 80°. This is not physiologically possible, as the total range of motion in the frontal plane is approximately 35° [56]. Nevertheless, this observation is consistent with reports by other authors on the Plug-in Gait model, in which only two markers are placed on the foot. This approach can lead to inaccuracies in ankle and foot kinematics [55], since at least three markers are assumed to be needed to track the kinematics of a rigid body at three degrees of freedom [57]. On the other hand, the HTC Vive Tracker, used in both eMotion protocols, acts as a rigid cluster equipped with an internal 9-DOF IMU and 18 infrared sensors placed at different angles, enabling precise real-time tracking of all six degrees of freedom [24,26]. The internal-sensor fusion algorithm combines inertial and optical data to accurately determine both the position and orientation of the tracker, making it sufficient for representing the motion of an individual segment. In addition, it is worth noting that the choice of calibration procedure can significantly affect the accuracy of the position of anatomical landmarks and, consequently, the measured features of human motion, such as joint angles during the execution of movements [57,58].

An analysis of the applicability of two different systems for measuring joint kinematics during exercise and the repeatability of the results did not show the superiority of the Plug-in Gait model with the Vicon system over a low-cost motion-tracking system based on virtual reality accessories. In the case of the Plug-in Gait model and the ISB 6DOF protocol, one advantage is that the collected data can be more useful for dynamic simulations (including model scaling and kinematic analysis). However, a disadvantage is the need for additional calibrations and preparation of the subject. The measurements conducted show that the Simplified 6DOF protocol is characterized by a repeatability comparable to that of other protocols, and its main advantages are the ease of performing measurements and the quick preparation of the subject, which can be a key factor when choosing a method for measuring kinematics in patients (for example, during physiotherapy) or participants in motion-based games, including virtual reality applications.

It is worth expanding the study to assess the values of this parameter on a larger sample, also taking into account other movement sequences, which would allow for the choosing of a protocol for specific purposes. Studying joint-angle discrepancies can help identify potential limitations of the system and highlight areas for further research and development. The discourse should also explore whether these discrepancies affect the system’s suitability for rehabilitation, sports science, or therapeutic applications. Analyzing their impact can provide valuable insights into the system’s effectiveness in various fields, such as sports and therapy. Additionally, it is important to consider how optimizing sensor installation techniques could improve measurement accuracy, thereby enhancing the system’s utility in clinical practice.

## 5. Conclusions

An analysis was conducted of the possibility of using two motion-tracking systems to record lower-limb joint kinematics during squatting, along with an analysis of the repeatability of the results. Tests conducted with the eMotion system, a low-cost tracking solution combined with proprietary software for data analysis, demonstrated its ability to collect full kinematic data for lower-limb joints during squats and to assess movement variability, offering practical applications in sports training and rehabilitation. Continuous tracker trajectories were obtained for all segments of the lower limb. The repeatability analysis of the obtained results showed similar and, in most joints, higher repeatability compared to a more advanced, expensive tracking system.

Despite obtaining valuable data, the study had some limitations, including the limited number of participants and specific testing conditions. It is worth noting that inconsistencies were observed in transverse and frontal-plane kinematics; thus, potential sources of these inaccuracies, such as sensor location, calibration discrepancies and environmental factors, should be investigated. Future research should involve a larger population, more diverse environmental conditions, and various physical exercises to verify our results. By introducing experimental groups with different lower-limb movement patterns, researchers were able to systematically verify the adaptability of the proposed method to complex lower-limb kinetic chain movements, thereby greatly enhancing the generalizability of the conclusions. An important limitation of the study is the lack of consideration of physiological variability among participants. The analysis does not take into account the distribution of physiological parameters, such as height, BMI, or limb length, which can affect movement dynamics and the measurement accuracy. Future research should focus on comparing tracking accuracy in subgroups with different physiological characteristics, as well as quantitatively assessing the effect of individual characteristics on measurement errors. It would also be beneficial to conduct tests with dummies, which would allow for an accurate assessment of the precision of kinematic value calculations using the proposed models.

## Figures and Tables

**Table 1:** Mean value of joint range of motion (ROM) during squats, calculated for all subjects and repetitions, along with their standard deviation.

Rotations	eMotion + Simplified 6DOF with Trackers	eMotion + ISB 6DOF with Trackers	Vicon + Plug-In Gait Model
Hip flex/ext	78.9° +/− 24.6°	96.1° +/− 24.6°	87.8° +/− 10.9°
Hip abd/add	22.3° +/− 6.8°	21.0° +/− 11.2°	19.2° +/− 9.6°
Hip int/ext	24.2° +/− 8.3°	24.2° +/− 18.1°	19.9° +/− 4.1°
Knee flex/ext	110.3° +/− 17.2°	104.9° +/− 34.8°	100.8° +/−15.1°
Knee abd/add	18.5° +/− 7.3°	26.4° +/− 23.8°	45.1° +/− 10.2°
Knee int/ext rotation	19.0° +/− 12.0°	54.9° +/− 44.4°	90.7° +/− 18.9°
Ankle dor/pla	28.7° +/− 5.5°	20.4° +/− 11.1°	33.1° +/− 7.2°
Ankle inv/ev	6.25° +/− 3.2°	12.3° +/− 5.2°	14.0° +/− 10.5°
Ankle abd/add	4.9° +/− 2.1°	14.0° +/− 6.8°	65.3° +/− 21.9°

**Table 2:** Differences between ROM values calculated using different protocols, along with their standard deviation.

Rotations	eMotion + ISB 6DOF with Trackers Compared with eMotion + Simplified 6DOF with Trackers	eMotion + ISB 6DOF with Trackers Compared with Vicon + Plug-In Gait Model	eMotion + Simplified 6DOF with Trackers Compared with Vicon + Plug-in Gait Model
Hip flex/ext	16.7° +/− 24.1°	9.0° +/− 8.5°	8.1° +/− 21.6°
Hip abd/add	0.8° +/− 15.6°	3.1° +/− 9.1°	2.4° +/− 17.6°
Hip int/ext	0.5° +/− 20.2°	4.4° +/− 21.3°	5.1° +/− 19.5°
Knee flex/ext	5.8° +/− 27.5°	8.2° +/− 18.2°	2.5° +/− 33.4°
Knee abd/add	9.0° +/− 22.1°	27.5° +/− 8.1°	18.6° +/− 26.5°
Knee int/ext rotation	38.1° +/− 49.3°	70.7° +/− 21.5°	35.4° +/− 39.6°
Ankle dor/pla	8.4° +/− 7.0°	5.4° +/− 7.0°	13.7° +/− 9.1°
Ankle inv/ev	5.7° +/− 6.0°	6.6° +/− 13.3°	0.7° +/− 12.0°
Ankle abd/add	9.3° +/− 6.2°	60.3° +/− 22.5°	50.9° +/− 26.5°

**Table 3:** Average inter-trial variability over the squat cycle across all subjects (present study) and the average inter-trial over the gait cycle as reported in the literature (previous studies).

Rotations [◦]	Present Study			Previous Study		
	eMotion + Simplified 6DOF with Trackers	eMotion + ISB 6DOF with Trackers	Vicon + Plug-In Gait Model	Simplified 6DOF with Trackers [25]	ISB 6DOF with Trackers [25]	Modified Helen Hayes Marker Set [32]
Hip flex/ext	3.09	3.58	1.73	1.56	1.36	1.2
Hip abd/add	1.23	1.22	1.60	0.91	0.89	0.5
Hip int/ext	1.45	1.39	4.63	1.63	1.44	1.2
Knee flex/ext	4.01	3.83	4.91	2.25	2.18	1.6
Knee abd/add	0.95	1.09	2.22	0.69	0.75	0.5
Knee int/ext rotation	1.34	2.20	4.75	1.32	1.37	1.2
Ankle dor/pla	1.39	1.18	3.66	1.62	1.59	1.3
Ankle inv/ev	0.83	0.92	0.84	1.26	1.22	-
Ankle abd/add	0.65	0.99	1.91	1.36	1.44	1.7

**Table 4:** F-test results for variance ratios: statistical significance of group differences (significant differences are shown in bold).

Rotations	eMotion + ISB 6DOF with Trackers Compared with eMotion + Simplified 6DOF with Trackers	eMotion + ISB 6DOF with Trackers Compared with Vicon + Plug-In Gait Model	eMotion + Simplified 6DOF with Trackers Compared with Vicon + Plug-In Gait Model
Hip flex/ext	*p* = 0.087	***p* < 0.001**	***p* < 0.001**
Hip abd/add	*p* = 0.248	***p* < 0.001**	***p* < 0.001**
Hip int/ext	*p* = 0.374	***p* < 0.001**	***p* < 0.001**
Knee flex/ext	*p* = 0.117	*p* = 0.083	*p* = 0.865
Knee abd/add	*p* = 0.437	***p* < 0.001**	***p* < 0.001**
Knee int/ext rotation	*p* = 0.437	***p* < 0.001**	***p* < 0.001**
Ankle dor/pla	***p* < 0.001**	***p* < 0.001**	***p* < 0.001**
Ankle inv/ev	*p* = 0.905	*p* = 0.08	*p* = 0.061
Ankle abd/add	***p* < 0.05**	***p* < 0.001**	***p* < 0.001**

## Data Availability

The dataset is available on request from the authors.
